# Potential Role of Acetyl-CoA Synthetase (*acs*) and Malate Dehydrogenase (*mae*) in the Evolution of the Acetate Switch in *Bacteria* and *Archaea*

**DOI:** 10.1038/srep12498

**Published:** 2015-08-03

**Authors:** Elliott P. Barnhart, Marcella A. McClure, Kiki Johnson, Sean Cleveland, Kristopher A. Hunt, Matthew W. Fields

**Affiliations:** 1 Department of Microbiology and Immunology, Montana State University, Bozeman, MT, USA; 2 Center for Biofilm Engineering, Montana State University, Bozeman, MT, USA; 3 US Geological Survey, Helena, MT, USA; 4 Department of Chemical and Biological Engineering, Montana State University, Bozeman, MT, USA; 5 Energy Research Institute, Montana State University, Bozeman, MT, USA; 6 ENIGMA, Lawrence Berkeley National Laboratory, Berkeley, CA, USA; 7 National Center for Genome Resources, Santa Fe, NM, USA

## Abstract

Although many *Archaea* have AMP-Acs (acetyl-coenzyme A synthetase) and ADP-Acs, the extant methanogenic genus *Methanosarcina* is the only identified Archaeal genus that can utilize acetate via acetate kinase (Ack) and phosphotransacetylase (Pta). Despite the importance of *ack* as the potential urkinase in the ASKHA phosphotransferase superfamily, an origin hypothesis does not exist for the acetate kinase in *Bacteria*, *Archaea*, or *Eukarya*. Here we demonstrate that Archaeal AMP-Acs and ADP-Acs contain paralogous ATPase motifs previously identified in Ack, which demonstrate a novel relation between these proteins in *Archaea*. The identification of ATPase motif conservation and resulting structural features in AMP- and ADP-acetyl-CoA synthetase proteins in this study expand the ASKHA superfamily to include acetyl-CoA synthetase. Additional phylogenetic analysis showed that Pta and MaeB sequences had a common ancestor, and that the Pta lineage within the halophilc archaea was an ancestral lineage. These results suggested that divergence of a duplicated *mae*B within an ancient halophilic, archaeal lineage formed a putative *pta* ancestor. These results provide a potential scenario for the establishment of the Ack/Pta pathway and provide novel insight into the evolution of acetate metabolism for all three domains of life.

The simplest alkane, one of the most abundant organic compounds on Earth, and the main component of natural gas, methane, has been a byproduct of microbial metabolism for over 3.46 Byr^1^. Methane production, carried out by methanogenic *Archaea* (methanogens), is an essential part of the global carbon cycle. Methanogens convert microbial metabolites into methane thereby allowing the bacterial breakdown of complex organic carbon compounds that would otherwise be thermodynamically unfavorable due to build-up of hydrogen and/or other metabolites^2^. Methanogens contain enzymes capable of producing methane via CO_2_ and H_2_ (hydrogenotrophic methanogenesis), one-carbon compounds (*e.g*., formate, methanol, methylamines and methylthiols) (methylotrophic methanogenesis) and/or acetate (aceticlastic methanogenesis)^3^. Acetate, a major microbial intermediate in anaerobic environments, can account for approximately two-thirds of the methane produced in the biosphere^4^,^5^,^6^,^7^.

The ability to utilize and/or excrete acetate to balance carbon and electron flow is wide-spread across the three domains of life, and this observation suggests that acetate utilization evolved early. The “acetate switch” refers to a metabolic capacity for cells to either excrete or assimilate acetate based upon the availability of nutrients^8^. This ability provides an adaptive advantage in dynamic environments by allowing rapid growth in the presence of abundant nutrients and enhanced survival when nutrients are depleted^8^. The proteins AMP acetyl-coenzyme A synthetase (AMP-Acs), ADP acetyl-coenzyme A synthetase (ADP-Acs) and a pathway involving acetate kinase (Ack) and phosphotransacetylase (Pta) are central to the acetate switch and catalyze the overall process of acetate production or acetate utilization^8^.

The Ack/Pta pathway is completely reversible and functions optimally at high concentrations of acetate that allows an adaptive advantage in dynamic environments where overflow metabolism occurs in the presence of abundant nutrients^9^,^10^. Although many *Archaea* have AMP*-acs* and ADP*-acs*, the extant methanogenic genus *Methanosarcina* is the only identified Archaeal genus with the Ack/Pta pathway and is the only known lineage to have all three genes (*i.e*., *ack*, AMP*-acs*, and ADP*-acs*). The absence of the Ack/Pta pathway in all other known *Archaea* indicates this pathway either evolved within an ancient archaeal genome or was horizontally transferred from another domain. Based upon the available sequences at the time, previous research suggested both *ack* and *pta* were provided to an ancient methanogen through horizontal gene transfer from *Bacteria*^11^. Although there is not an origin hypothesis for *ack* (or *pta*) in *Bacteria*, an alternative hypothesis has not been proposed because *ack* or *pta* homologs have not been identified in any other *Archaea*. Here we demonstrate that Archaeal AMP-Acs and ADP-Acs contain paralogous ATPase motifs previously identified in Ack, revealing novel Ack homologs in *Archaea*. Furthermore, a Pta homolog with presumptive active site residues was identified within halophilic *Archaea* (*e.g*., *Halobacteriales*) that have been previously shown to exchange DNA with phylogenetically disparate relatives^12^,^13^. These observations provide a possible scenario for the transfer of *pta* to an ancient halophilic methanogen that possessed *ack* and the establishment of the Ack/Pta pathway. The presented results coincide with the evolution of the first protein kinase as Ack that was previously postulated to be the urkinase and the most ancient protein in the large ASKHA (acetate and sugar kinases/Hsc70/actin) superfamily of phosphotransferases that share an ATPase domain through gene duplication and divergence^14^,^15^. Recent work indicated that a brief period of the Archaeon eon coincided with rapid diversification of bacterial lineages that gave rise to 27% of modern gene families as a consequence of duplication and divergence of genes involved in cellular metabolism^16^. The evolution of *ack* and the development of the ASKHA superfamily may have contributed to the early metabolic expansion in biology identified in the Archaeon time period.

## Results

### ATPase Motif Conservation

**Figure 1:**
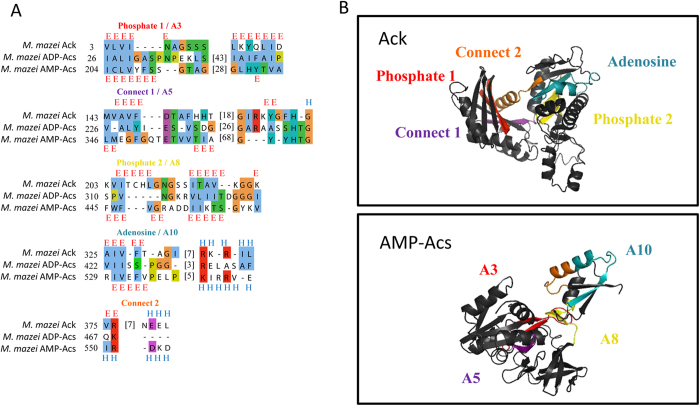
(**A**) *Methanosarcina* AMP-Acs, ADP-Acs and Ack multiple alignment highlighting the shared motifs. The previously identified motifs in Ack and AMP-Acs are above the alignment along with the secondary structure of Ack. Residues are colored according to ClustalX and dashes indicate gaps^59^. The secondary structure of AMP-Acs is labeled below the alignment. (**B**) Structural comparisons between Ack and AMP-Acs with previously identified motifs highlighted and color coded^25^,^26^.

The presented results demonstrate that AMP-Acs, ADP-Acs and Ack share motif and active site features (Fig. 1A). The active site of AMP-Acs, located between the two domains of the protein, is formed by core motifs that include a serine and glycine-rich loop (termed region A3), a sequence that contains a threonine-glutamate dipeptide with an invariant glutamate residue (A5), a DX6GXR motif with an invariant glycine (A8), and a region that contains a lysine residue completely conserved in all AMP-forming enzymes (A10)^17^,^18^. The ADP-Acs active site has not been well defined due to a lack of protein structures but active site and motif inferences have been previously reported based on a close relationship to succinyl-CoA synthetase (SuccCoA) proteins^19^,^20^,^21^. The α- and β-subunits of ADP*-*Acs and SuccCoA are often either fused or closely associated ensuring that these subunits are transcribed and work in tandem^20^,^22^,^23^. The ADP-Acs and SuccCoA α−subunits contain a motif similar to the A3 motif in AMP-Acs and an active site dependent on the phosphorylation of a conserved histidine and glutamate residue that aligns with the A5 region in AMP-Acs (Fig. 1A)^19^. Similar to AMP-Acs and ADP-Acs, Ack is a two domain protein with an initial serine and glycine rich motif (termed Phosphate 1)^14^,^24^. The second motif (Connect 1) aligns with A5 in AMP-Acs, with an invariant negatively charged glutamate in place of aspartate in both ADP-Acs and AMP-Acs. An active site histidine residue is completely conserved in Ack, ADP-Acs, SuccCoA and several AMP-Acs^20^,^25^,^26^. In addition, the phosphate 2 motif and the adenosine motif in Ack share homology and secondary structural features with the A8 and A10 motifs from AMP-Acs respectively (Fig. 1A)^17^,^24^.

A HMM-HMM comparison^27^ with a full alignment (Supp. Fig. 1) identified significant homology between the AMP-Acs, ADP-Acs, SuccCoA synthetase, butyrate kinase (Buk), and Ack sequences. HHPred^28^ predicted Ack, AMP-Acs and ADP-Acs as homologs with high confidence based on structures and folds (E-values < 1E^-^12) according to both HMM-HMM global and local multiple alignment comparisons. The three dimensional structures of AMP-Acs, ADP-Acs and Ack indicate that all three proteins consist of two domains^17^,^20^,^29^. The two domains join together forming the active site cleft with an ATP-binding site at the bottom, a feature shared by all proteins in the ASKHA superfamily. This structural feature indicates all of the proteins in the ASKHA superfamily were likely formed through gene duplication and divergence because the structure is the result of complex internal core folds due to motif conservation involved with ATP binding^15^,^30^. Previous identification of these structural features in Ack suggested this protein was the ancestral protein of the ASKHA superfamily and potentially the first kinase protein that evolved (Urkinase)^14^,^30^. The structural comparisons in Fig. 1B provided the ability to calculate the root-mean-square distance (RMSD) values for the motifs shared between AMP-Acs and Ack (Table 1). The identification of ATPase motif conservation and resulting structural features in AMP- and ADP-acetyl-CoA synthetase proteins in this study expand the ASKHA superfamily to include acetyl-CoA synthetase (Fig. 1).

### Phylogenetic Relationships

**Figure 2:**
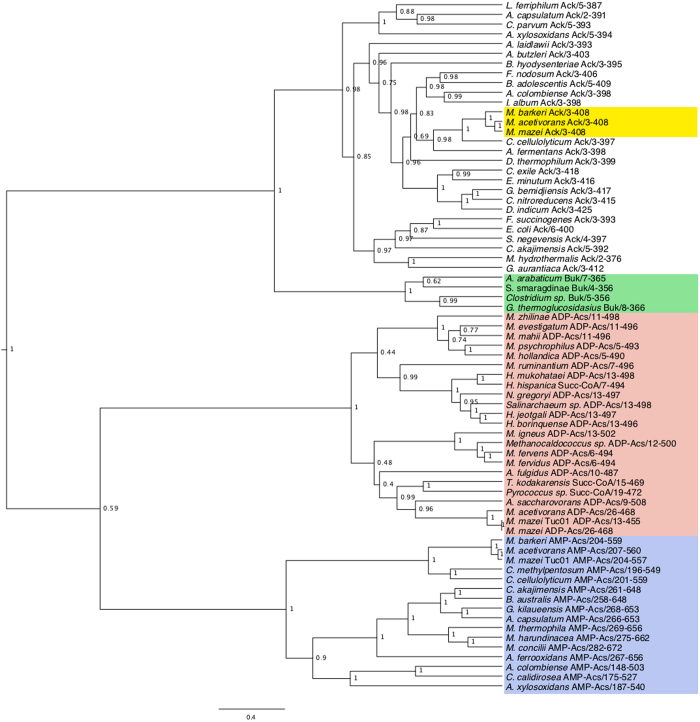
Phylogenetic analysis of Buk, Ack, SuccCoA, ADP-Acs and AMP-Acs using BEAST. The posterior probabilities label each node and branch lengths are scaled to expected substitutions per site. The yellow box denotes *Methanosarcina* Ack sequences, the green box denotes Buk sequences, the red box denotes ADP-Acs sequences, and the blue box denoted AMP-Acs sequences.

A phylogenetic comparison of Ack, AMP-Acs, ADP-Acs and SuccCoA shows the evolutionary relationship of the proteins (Fig. 2). Branch lengths indicate a large number of substitutions between Ack and both ADP-Acs and AMP-Acs. Buk and Ack appear to share a common ancestor, and this result supports previous research that suggested Buk evolved from an ancient Ack^14^. The results demonstrate homology for AMP-Acs, ADP-Acs and Ack that suggests these acetate-metabolizing proteins share a common ancestor.

### Genome Synteny Investigations

**Figure 3:**
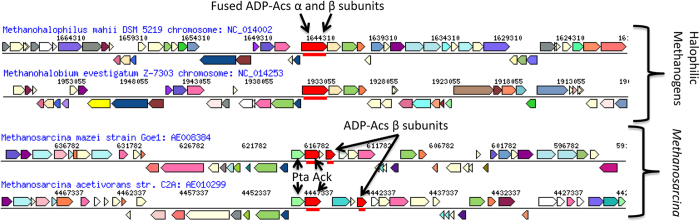
Genome alignment between *Methanosarcina* and halophilic methanogens. Halophilic methanogens fused ADP-Acs a and b subunits (red) exhibit synteny to *ack* (red) and the ADP-Acs b subunit (red) in *Methanosarcina mazei* and *Methanosarcina acetivorans*. The *pta* (light green) is closely associated with *ack* within all sequenced *Methanosarcina* genomes. The color shading indicates homology shared between the genomes.

*Methanosarcina* spp. are the only identified *Archaea* with Ack, and this observation suggests *ack* was either horizontally transferred or could have evolved within the archaeal genomes^11^. Genome synteny can be maintained for hundreds of millions of years, therefore genes of close phylogenetic relatives in the same loci are frequently orthologous^31^. Genome synteny comparisons suggest *ack* in *Methanosarcina* is located in the same locus as the gene encoding the *ADP-acs* α subunit in the close phylogenetic relatives *M. mahii* and *M. evestigatum* (Fig. 3). Furthermore, *ack* in *Methanosarcina spp*. is located near the ADP*-acs* β-subunit while in most other Archaeal genomes, including *M. mahii* and *M. zhilinae*, the genes encoding the α- and β-subunits of ADP*-*Acs are either fused or closely associated^22^,^23^. As demonstrated in Fig. 3, the *ack* and ADP*-acs* (β−subunit) genes are in the same locus (separated by 1 or 2 genes) in the *Methanosarcina mazei* and *Methanosarcina acetivorans* genomes. *M. mazei* and *M. acetivorans* contain a sole gene encoding the *ADP-acs* α−subunit (gi20904723 and gi19919192) in a gene locus different from the halophilic methanogens and separate from the gene encoding the β-subunit of ADP-Acs and these results support a possible scenario for the evolution of *ack* (see discussion).

### Evolution of a Catalytic Pta in *Halobacteriales*

**Figure 4:**
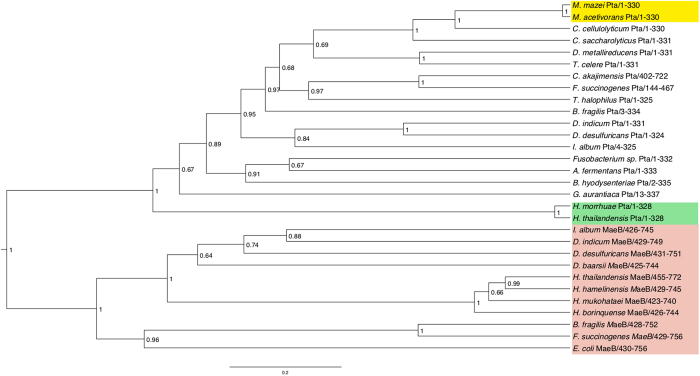
Phylogenetic relationships of archaeal and bacterial Pta and Ack sequences via BEAST analysis. The yellow box denotes *Methanosarcina* Pta sequences, the green box denotes haloarchaeal Pta sequences, and the red box denotes MaeB sequences.

**Figure 5:**
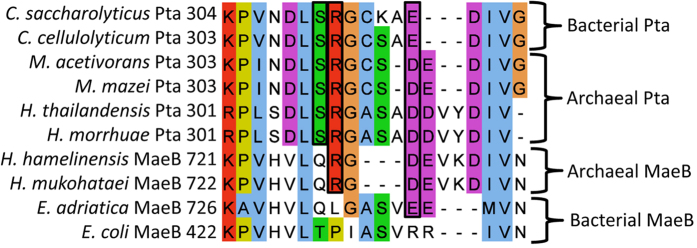
Alignment highlighting the shared active site residues between archaeal and bacterial MaeB and Pta. Active site residues are in black boxes. Sequences are colored according to the ClustalX color scheme and dashed indicate gaps in the alignment.

Pta reversibly exchanges CoA and phosphate groups between acetyl-CoA and acetyl-P, maintaining a balance between biosynthesis and energy generation^32^,^33^. The presented analyses identified two different Pta isoforms (Pta and MaeB) in *Halobacteriales* not previously described in *Archaea* (Fig. 4). MaeB consists of a fusion between the malic enzyme and Pta that has been characterized in *Bacteria*^34^. Results suggest the homologous Pta portion of MaeB enables better metabolic modulation of malic enzyme but lacks Pta activity^34^. Many *Halobacteriales* contain duplicate *mae*B genes providing the possibility for one copy to diverge. *Halococcus* spp. contain *maeB* as well as a presumptive *pta* that contains the conserved catalytic residues^35^; however, further work is needed to confirm Pta activity. Phylogenetic analysis showed that Pta and MaeB sequences had a common ancestor, and that the Pta lineage within the halophilc archaea was an ancestral lineage to the bacterial and *Methanosarcina* sequences (Fig. 4). These results suggest that divergence of a duplicated *mae*B within the ancient *Halococcus* lineage could have formed a putative *pta* ancestor. A conserved Pta residue (R310) was present in all *Halobacteriales* MaeB but absent in bacterial MaeB analyzed in this report (Fig. 5)^35^. The haloarchaeal Pta sequences were more closely related to those of *Methanosarcina* and *Bacteria*, and further work is needed to discern the relationship and significance of the homologous regions of archeal Pta and MaeB sequences.

## Discussion

Our results provide a novel link between AMP-Acs, ADP-Acs and Ack through duplication and divergence of shared motifs involved in ATP binding. The major high-transfer potential molecule of the cell, ATP, is critical for all extant life on Earth that is currently known. It is suggested that deep within hydrothermal vents, AMP-Acs evolved the ability to bind ATP through bio-mimicry of the mineral greigite^36^. The notion of divergent evolution for the Ack has been previously postulated, and the ancestral ATPase has evolved into a diverse set of proteins across all three domains with a myriad of functions (*e.g*., actin, hexokinase, hsp70)^8^. Bork *et al*. argued it unlikely that the rather particular and complicated fold of the ATPase domain would arise twice as result of evolutionary pressure^11^; and therefore, the relationship is likely a result of divergent evolution. Providing a relationship between AMP-Acs, ADP-Acs, and Ack allows deeper insight into the evolution of the ASKHA superfamily and an ATPase domain common to proteins in all three domains of life, including actin in eukaryotic cells^14^,^15^.

Genome synteny results suggest a gene encoding an ADP-Acs α subunit could have duplicated and diverged into a gene which encodes Ack within an ancient *Archaeon*. The ADP-Acs α subunit from halophilic methanogens has features including a two domain structure, a glutamate residue that aligns with the magnesium binding aspartate in Ack, and a conserved C-terminus glycine residue that was predicted to be part of the ancient Ack by other researchers^14^,^15^. In addition, the evolution of a Pta in *Halobacteriales* could have allowed horizontal gene transfer of a duplicated *pta* to an ancient halophilic methanogen with *ack* and the establishment of the first Ack/Pta pathway. This scenario is supported by research that indicates halophilic methanogens and *Halobacteriales* engage in genetic exchange similar to conjugation in *Bacteria*^37^,^38^.

The high identity of the archaeal Ack and Pta to homologs in several *Eukarya* and many *Bacteria* suggests a single ancient origin of the Ack/Pta pathway^39^. The presence of biogenic methane in approximately 3.5-Gyr-old fluid inclusions, when the oceans were 1.6 times saltier than today, indicate halophilic methane production and halophiles evolved early^1^,^40^. An early reliance on sodium may have promoted co-localization of halophilic methanogens that possessed the presumptive *pta* and *ack* genes with ancient *Bacteria* that relied on F-type and/or V-type ATPases for energy production^40^. Phylogenetic analysis suggests *pta* and *ack* in *Methanosarcina* are most closely related to those in different *Clostridium* species^11^. The *ack* and *pta* in *Clostridium* are in a locus different from the gene cluster for the ancient Wood-Ljungdahl pathway^41^ and in the same orientation as the homologs observed in *Methanosarcina* which implicates horizontal gene transfer of both genes in one transfer event between the ancient *Methanosarcina* and *Clostridium*. The presented evolutionary relationship between ADP-Acs, AMP-Acs, and Ack provides an evolutionary path for the development of Ack that was then shared between *Methanosarcina* and *Clostridium*. The extensive distribution of *ack* throughout *Bacteria* and *Eukarya* suggests Ack contributed to the evolutionary fitness of many organisms across all domains of life^11^.

Today, many organisms take advantage of the reversibility of the Ack/Pta pathway to conserve energy and maintain steady-state levels of free CoA in the cell^42^. This flexibility contributes greatly to cell survival when alternating between copiotrophic and oligotrophic conditions often experienced *in situ*^8^. Thermodynamic constraints of acetate activation suggest Ack/Pta is suboptimal for acetate consumers at low acetate concentrations, while these organisms would have an increased energetic yield in high acetate environments. Conversely for acetate producing organisms, Ack/Pta provides a thermodynamic gradient which is functional under the largest range of acetate concentrations whereas AMP-Acs is often considered irreversible. During direct resource competition, increased rate or alternative substrate utilization can provide a major competitive advantage^43^.

Research indicates that a brief period of the Archaean eon coincided with rapid diversification of bacterial lineages that gave rise to 27% of major modern gene families, and functional analyses of the genes originating during this expansion show likely involvement in electron-transport and respiratory pathways^16^. The expansion could have contributed to the wide distribution of the Ack/Pta pathway in many bacterial lineages and the evolution of the ASKHA superfamily^14^. Additionally, eukaryotes have incorporated Ack into at least three different metabolic pathways including the Ack/Pta pathway, the Ack/Xfp pathway and a pathway involving ADP-Acs and Ack^44^. Ack has been observed to be widespread in fungi with putative *ack* sequences identified in three of the four fungal phyla including the *Chytridiomycota*, the earliest branch of true fungi^44^. Although the evolution of the Ack/Pta pathway may have greatly expanded the diversity of life on Earth, the enhanced methane production provided by the pathway (*i.e*., acetoclastic methanogenesis) under certain environmental conditions may have greatly decreased diversity at least once.

Five major extinction events have been identified that greatly diminished the diversity of life on Earth^45^. Previous research suggests the largest extinction, the end-Permian extinction that decimated up to 95% of shell-bearing marine species and 80% of land animals, was the result of a large release of biogenic methane^46^,^47^. The presented results with additional genome sequences suggest that a Ack/Pta pathway could have been acquired or evolved within a methanogenic genome. Thus, the potential for a large methane release was in place long before the end-Permian extinction, suggesting a change in environmental conditions could have promoted methanogenic microorganisms. Recent research indicates that the ancient combustion of coal through an interaction with basalt sills of the Siberian Traps released trace metals that created stimulatory conditions for methane production and instigated the end-Permian extinction^47^,^48^,^49^,^50^. Today, atmospheric methane concentrations have nearly tripled since pre-industrial times and this increase has been partly attributed to coal combustion^51^,^52^.

The presented data offers the first evolutionary relationships between *acs* and *ack* as well as *mae* and *pta* and has implications for the development of protein kinases and the Ack/Pta pathway. The presented results reveal novel motif, active site, and structural conservation between AMP-Acs, ADP-Acs and Ack that suggest the proteins are related through gene duplication and divergence. A possible scenario is suggested in which the first Ack could have evolved from a duplicated ADP-Acs α-subunit in an ancestral *Archaeon*. A *pta* homolog is conserved in all the *Halobacteriales* genomes sequenced to date, and the close relation of *Methanosarcina spp*. to halophilic methanogens suggests the *pta* gene could have been acquired via horizontal gene transfer from ancient *Halobacteriales* that existed in the same environment. This event greatly impacted the cycling of carbon via a metabolic node that coordinates cellular catabolism and anabolism, and likely was a turning point in the evolutionary history of microbial life and carbon cycling on a global scale. For example, access to the acetate switch would have allowed greater energy conservation that likely played a role in early cell development^53^. An increased understanding of the Ack/Pta pathway origins and the historical impacts on the biosphere will provide insight into the evolution and diversification of biological function in diverse microbial communities.

## Methods

### Sequence Alignment

Amino acid sequences: *L. ferriphilum* Ack gi406774110 *A. capsulatum* Ack gi506377611, *C. parvum* Ack gi193086734, *A. xylosoxidans* Ack gi310762969, *A. laidlawii* Ack gi161985465, *A. butzleri* Ack gi526474464, *B. hyodysenteriae* Ack gi225619127, *F. nodosum* Ack gi154153381, *B. adolescentis* Ack gi740659093, *A. colombiense* Ack gi293617041, *I. album* Ack gi383801907, *M. barkeri* Ack gi73669326, *M. acetivorans* Ack gi20092406, *M. mazei* Ack gi17907861, *C. cellulolyticum* Ack gi506405859, *A. fermentans* Ack gi502703469, *D. thermophilum* Ack gi501541912, *C. exile* Ack gi381363692, *E. minutum* Ack gi501383324, *G. bemidjiensis* Ack gi197088018, *C. nitroreducens* Ack gi312939484, *D. indicum* Ack gi503271010, *F. succinogenes* Ack gi261372347, *E. coli* Ack gi15802843, *S. negevensis* Ack gi503709960, *C. akajimensis* Ack gi293614294, *M. hydrothermalis* Ack gi328451244, *G. aurantiaca* Ack gi226090818, *A. arabaticum* Buk gi302391242, *S. smaragdinae* Buk gi301637019, *Clostridium* sp. Buk gi357173877, *G. thermoglucosidasius* Buk gi336234763, *M. zhillinae* ADP-Acs gi335930152, *M. evestigatum* ADP-Acs gi298675798, *M. mahii* ADP-Acs gi294496302, *M. psychrophilus* ADP-Acs gi410670387, *M. hollandica* ADP-Acs gi505136916, *M. ruminatium* ADP-Acs gi502721385, *H. mukohataei* ADP-Acs gi506243470, *H. hispanica* ADP-Acs gi503806362, *N. gregoryi* ADP-Acs gi491747964, *Salinarchaeum* sp. ADP-Acs gi510882650, *H. jeotgali* ADP-Acs gi495691923, *H. borinquense* ADP-Acs gi313126866, *M. igneus* ADP-Acs gi503565020, *Methanocaldococcus sp*. ADP-Acs gi502746162, *M. fervens* ADP-Acs gi506272139, *M. fervidus* ADP-Acs gi312137221, *A. fulgidus* ADP-Acs gi668356014, *T. kodakarensis* SuccCoA gi57160139, *Pyrococcus* sp. SucSoA gi389853130, *A. saccharovorans* ADP-Acs gi302349008, *M. acetivorans* ADP-Acs gi19917192, *M. mazei* Tuc01 ADP-Acs gi452208966, *M. mazei* ADP-Acs gi21226460, *M. barkeri* AMP-Acs gi73669665, *M. acetivorans* AMP-Acs gi20091733, *M. mazei* Tuc1 AMP-Acs gi452211690, *C. methylpentosum* AMP-Acs gi493399067, *C. cellulolyticum* AMP-Acs gi220928894, *C. akajimensis* AMP-Acs gi754108219, *B. australis* AMP-Acs gi505211453, *G. kilaueensis* AMP-Acs gi752573627, *A. capsulatum* AMP-Acs gi225791282, *M. thermophila* AMP-Acs gi116666161, *M. harundinacae* AMP-Acs gi386001446, *M. concilii* AMP-Acs gi330506788, *A. ferrooxidans* AMP-Acs gi753964065, *A. colombiense* AMP-Acs gi502814190, *C. calidirosea* AMP-Acs gi512725278, *A. xylosoxidans* AMP-Acs gi503158389, *M. mazei* Pta gi452099000, *M. acetivorans* Pta gi19917662, *C. cellulolyticum* Pta gi220929552, *C. saccharolyticus* Pta gi346722880, *D. metallireducens* Pta gi493768054, *T. celere* Pta gi517490922, *C. akajimensis* Pta gi502808194, *F. succinogenes* Pta gi504358929, *T. halophilus* Pta gi503890526, *B. fragilis* Pta gi53711769, *D. indicum* Pta gi503271009, *I. album* Pta gi383801098, *Fusobacterium* sp. Pta gi696260554, *A. fermentans* Pta gi502703540, *B. hyodysenteriae* Pta gi501921082, *G. auranitica* Pta gi226090483, *H. morrhuae* Pta gi490155740 *H. thailandensis* Pta gi495013868, *I. album* MaeB gi504373553, *D. indicum* MaeB gi503270289, *D. desulfuricans* MaeB gi502772806, *D. baarsil* MaeB gi760132925, *H. thailandensis* MaeB gi495015987, *H. hamelinensis* MaeB gi445789056, *H. mukohataei* MaeB gi257170109 *H. borinquense* MaeB gi313125645, *B. fragilis* MaeB gi695330578, *F. succinogenes* MaeB gi504358448, *E. coli* MaeB gi378259043 were obtained from the National Center for Biotechnology Information (NCBI) genomic database. The sequences were trimmed and aligned with Mafft using a default setting followed by the settings E-INS-I and finally L-INS-I^54^,^55^. The sequences were manually adjusted based on secondary structures provided by Jpred^56^. Sequences were colored according to ClustalX with dashes indicating gaps^57^. Using JGI-IMG Usearch and Nearest Neighbor functions, a gene synteny map was generated for *Methanosarcina* Ack (gi17907861)^58^,^59^. In an iterative fashion we investigated the synteny in related halophilic methanogens.

### HHPred Analysis

Using HHPred^28^ (Open source 2.0) a homology detection and structure predictor by HMM-HMM comparison 6 sequences (2AMP-Acs, 2ADP-Acs, 2Ack) from our alignment were analyzed by HHPred. Using the HHBlits module at default parameters in global alignment mode these sequences were compared to the PDB70 HMM database^28^. The structural comparison results indicated ADP-Acs, AMP-Acs, Ack and Buk were homologous at a probability of 100% with e-values of 9.1E-82, 8.4E-63, 1.3E-23, 1E-12, respectively. This structural evidence supports homology at the superfamily and fold level. Using default parameters but different databases such as CDD, Interpro andPfam^28^, the outcome resulted with lower e-values and better probability due to the availability of more sequenced data.

### Pymol Analysis

The structure AMP-Acs *M. acetivorans* (3ETC_A) and Ack *M. thermophila* (1g99) were downloaded from the protein database (PDB)^28^. All structures were trimmed to represent the multiple alignment. The motifs which form the ATPase cleft were identified with Pymol. (The PyMOL Molecular Graphics System, Version 1.5.0.4 Schrödinger, LLC).

### Phylogenetic Analysis

A Bayesian BEASTv1.8.0^60^ tree was created from the manually adjusted sequence alignment using a 10 million step chain with 20% burn-in; run under the WAG amino acid substitution model^61^ and rate heterogeneity among sites (gamma distribution with 8 categories) with relaxed exponential clock model and coalescent constant population tree prior. Four Constraint priors were applied for the Buk, Ack, AMP-Acs and ADP-Acs groups from a preliminary UPGMA tree generated from Molecular Evolutionary Genetics Analysis (MEGA)^62^ (data not shown); a randomized tree was used as the starting tree input.

## Additional Information

**How to cite this article**: Barnhart, E. P. *et al*. Potential Role of Acetyl-CoA Synthetase (*acs*) and Malate Dehydrogenase (*mae*) in the Evolution of the Acetate Switch in *Bacteria* and *Archaea*. *Sci. Rep*. **5**, 12498; doi: 10.1038/srep12498 (2015).

## Supplementary Material

Supplementary Information

## Figures and Tables

**Table 1:** Comparison between previously identified Ack and AMP-Acs motifs with resulting RMSD values^8^,^15^.

Ack Motif	AMP-Acs Motif	RMSD Value
Phosphate 1	A3	1.82
Connect 1	A5	3.72
Phosphate 2	A8	2.18
Adenosine	A10	1.19
Connect 2	C-terminus	2.76
