# Supplementary material for: Potential Role of Acetyl-CoA Synthetase (acs) and Malate Dehydrogenase (mae) in the Evolution of the Acetate Switch in Bacteria and Archaea
Source: Sci Rep. 2015 Aug 3;5:12498. doi: 10.1038/srep12498 (PMC4522649; doi:10.1038/srep12498)

Title: **Role of Acetyl-CoA Synthetase (*acs*) and Malate Dehydrogenase (*mae*) in the Evolution of the Acetate Switch in *Bacteria* and *Archaea***

Contributors: Elliott P. Barnhart[1,2,3], Marcella A. McClure[1], Kiki Johnson[1], Sean Cleveland[1], Kristopher A. Hunt[2,4] and Matthew W. Fields[*1,2,5,6,7]

Affiliations:
[1]Department of Microbiology and Immunology, Montana State University, Bozeman, MT
[2]Center for Biofilm Engineering, Montana State University, Bozeman, MT
[3]U.S. Geological Survey, Helena, MT
[4]Department of Chemical and Biological Engineering, Montana State University, Bozeman, MT
[5]Energy Research Institute, Montana State University, Bozeman, MT
[6]ENIGMA (http://enigma.lbl.gov/)
[7]National Center for Genome Resources, Santa Fe, NM

Running Title: Evolution of Acetate Kinase

Key Words: Methanogen; *Methanosarcina*; phosphotransaceytlase (pta)

Corresponding Author:
Dr. M.W. Fields[*]
Department of Microbiology and Immunology
Center for Biofilm Engineering
366 EPS Building
Montana State University
Bozeman, MT 59717
406-994-7340
matthew.fields@biofilm.montana.edu

Supplementary Figure: Full alignment of representative protein sequences analyzed to infer conservation between AMP-acetyl coA synthetase (AMP-Acs), succinyl CoA (SucCoA), ADP-acetyl CoA synthetase (ADP-Acs), acetate kinase (Ack), and butyrate kinase (Buk).

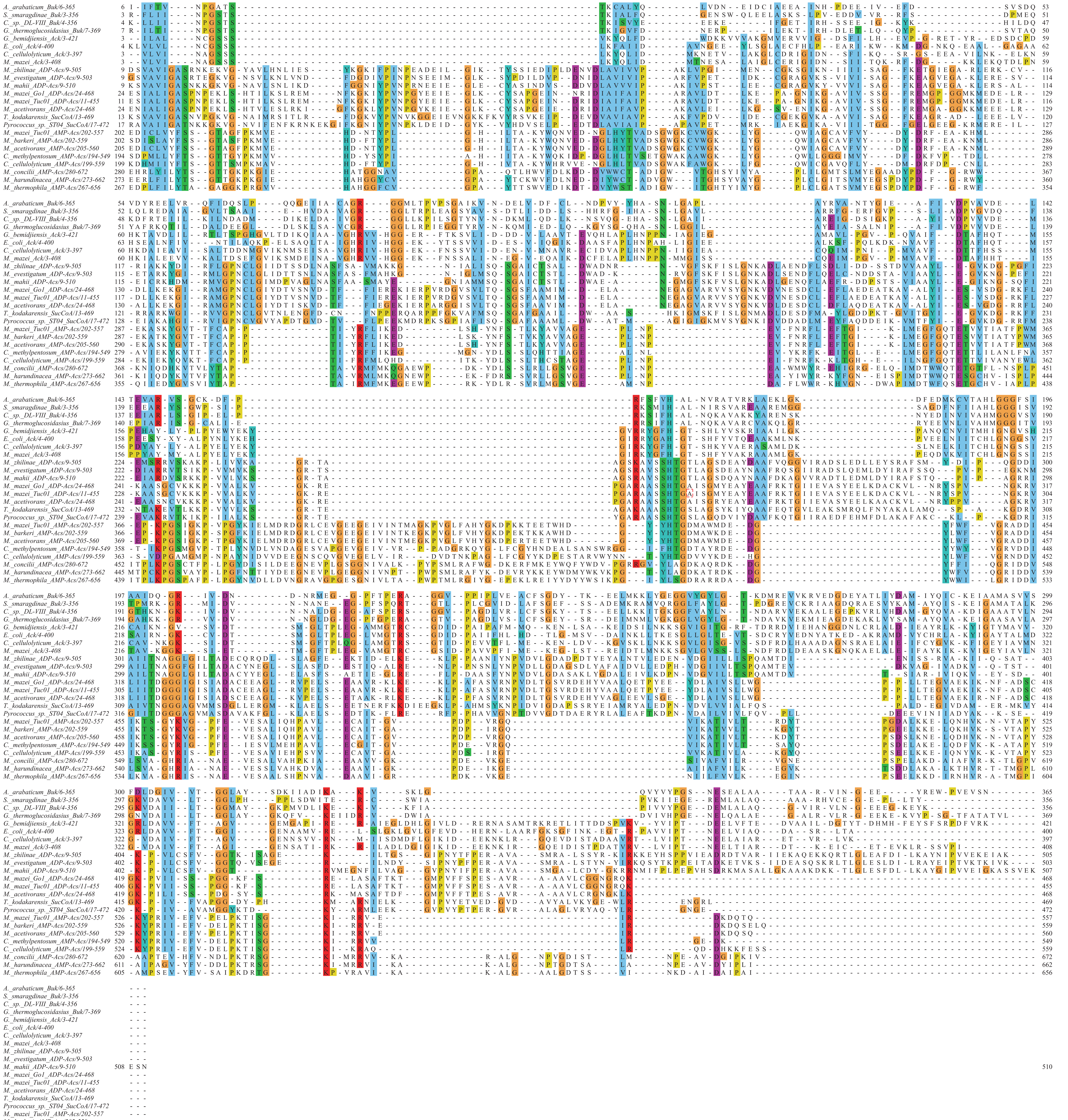

Supplement: Supplementary Information [file srep12498-s1.pdf]
